# Identifying potential areas of expansion for the endangered brown bear (*Ursus arctos*) population in the Cantabrian Mountains (NW Spain)

**DOI:** 10.1371/journal.pone.0209972

**Published:** 2019-01-04

**Authors:** Alejandra Zarzo-Arias, Vincenzo Penteriani, María del Mar Delgado, Paloma Peón Torre, Ricardo García-González, María Cruz Mateo-Sánchez, Pablo Vázquez García, Fredrik Dalerum

**Affiliations:** 1 Research Unit of Biodiversity (UMIB, UO-CSIC-PA), Oviedo University—Campus Mieres, Mieres, Spain; 2 Pyrenean Institute of Ecology (IPE), CSIC, Zaragoza, Spain CSIC, Zaragoza, Spain; 3 Consejería de Ordenación del Territorio, Infraestructuras y Medio ambiente, Dirección General de Biodiversidad, Principado de Asturias, Oviedo, Spain; 4 Pyrenean Institute of Ecology (IPE), CSIC, Jaca, Spain; 5 ECOGESFOR Research Group, E.T.S.I Montes, Forestal y del Medio Natural, Technical University of Madrid, Ciudad Universitaria s/n, Madrid, Spain; 6 Instituto Cavanilles Institute of Biodiversity and Evolutionary Biology, Universidad de Valencia, Valencia, Spain; 7 Department of Zoology, Stockholm University, Stockholm, Sweden; 8 Mammal Research Institute (MRI), Department of Zoology and Entomology, University of Pretoria, Hatfield, Pretoria, South Africa; National and Kapodistrian University of Athens, GREECE

## Abstract

Many large carnivore populations are expanding into human-modified landscapes and the subsequent increase in coexistence between humans and large carnivores may intensify various types of conflicts. A proactive management approach is critical to successful mitigation of such conflicts. The Cantabrian Mountains in Northern Spain are home to the last remaining native brown bear (*Ursus arctos*) population of the Iberian Peninsula, which is also amongst the most severely threatened European populations, with an important core group residing in the province of Asturias. There are indications that this small population is demographically expanding its range. The identification of the potential areas of brown bear range expansion is crucial to facilitate proactive conservation and management strategies towards promoting a further recovery of this small and isolated population. Here, we used a presence-only based maximum entropy (MaxEnt) approach to model habitat suitability and identify the areas in the Asturian portion of the Cantabrian Mountains that are likely to be occupied in the future by this endangered brown bear population following its range expansion. We used different spatial scales to identify brown bear range suitability according to different environmental, topographic, climatic and human impact variables. Our models mainly show that: (1) 4977 km^2^ are still available as suitable areas for bear range expansion, which represents nearly half of the territory of Asturias; (2) most of the suitable areas in the western part of the province are already occupied (77% of identified areas, 2820 km^2^), 41.4% of them occurring inside protected areas, which leaves relatively limited good areas for further expansion in this part of the province, although there might be more suitable areas in surrounding provinces; and (3) in the eastern sector of the Asturian Cantabrian Mountains, 62% (2155 km^2^) of the land was classified as suitable, and this part of the province hosts 44.3% of the total area identified as suitable areas for range expansion. Our results further highlight the importance of increasing: (a) the connectivity between the currently occupied western part of Asturias and the areas of potential range expansion in the eastern parts of the province; and (b) the protection of the eastern sector of the Cantabrian Mountains, where most of the future population expansion may be expected.

## Introduction

As a consequence of the implementation of major conservation and management actions [1,2], many large carnivore populations are expanding into human-modified landscapes [3–7], which may provoke an increase in several types of conflicts, e.g., livestock predation, crop damage and, more rarely, attacks on humans [8–10]. Predicting potential range expansion areas is an important step towards proactive management strategies minimizing conflict, thereby enhancing large carnivore population viability [11–13]. This is particularly important for small and isolated populations that are confined as a result of the expansion of humans and habitat degradation, and for which spatial expansion is therefore essential for their conservation. Different habitat suitability models, like maximum entropy models [14–16], are nowadays commonly used for exploring the availability of favourable habitats and the likely spatial distribution of population expansions, as well as the environmental factors determining them [17–20]. Since reliable absence data is frequently difficult to obtain, these models are usually based on species presence-only occurrence data such as maximum entropy models. In human-dominated landscapes, where fragmentation and loss of good habitat, primarily large patches of continuous forest with little human encroachment [21–23], is continually arising, these models have become very popular in carnivore population studies and conservation [24–27], given the abovementioned difficulty in obtaining absence data [28]. Further, a key ingredient of these models is the spatial scale considered. While large-scale spatial models are useful for understanding broad population patterns and processes related to the distribution of a species, high-resolution models offer information on specific niche requirements of locally adapted populations [29,30]. Thus, combining different spatial scales provides the opportunity to address and improve our knowledge about the relationships between species and the environment by providing more accurate predictions on species distributions [22,31].

The brown bear *Ursus arctos* is one of the most widespread large carnivores in the world, occupying different countries in North America, Europe and the north of Asia (http://www.iucnredlist.org/details/41688/0). Brown bears were historically persecuted and nearly eliminated from much of Western Europe in the 20^th^ century in order to avoid conflicts and as a result of hunting [5]. The effects of direct persecution were aggravated by other threats like habitat loss and fragmentation due to the expansion of the human population, which conflicts with the large spatial requirements of this species [32].

Northern Spain is home to the last two isolated populations of brown bear in south-western Europe, which have been protected for more than 30 years. The main population, which is estimated to consist of approximately 200 individuals (95% CI: 183–278; [33]) only, inhabits the Cantabrian Mountains (NW Spain) and is divided into two tenuously connected subpopulations [34], with most of the population inhabiting the western sector of the Cantabrian Mountains belonging to the province of Asturias (hereafter referred to as “Asturias”). Previous studies have shown that both subpopulations are increasing in number and range, especially in the western region [33–37]. This current positive trend may cause re-colonisations of areas where bears have disappeared and/or the occupation of new ones [3].

The small number of bears, their complete isolation and the limited connectivity between the two subpopulations make the study of the range expansion of this population particularly interesting. In addition, as its expansion might increase conflicts with human populations and activities (i.e. livestock predations, crop and apiary damages, attacks on humans), the identification of potential new colonization areas is of great importance for supporting conservation management actions. Here, by using maximum entropy models, we aim to identify the potential range expansion areas of Cantabrian brown bears. We have specifically: (1) identified suitable areas for bears at a coarse scale (5 x 5 km); (2) explored, at a finer scale (1 x 1 km), the best areas highlighted in the previous model; and (3) evaluated which environmental variables determine habitat suitability for this population on both scales.

## Materials and methods

### Study area

Asturias is one of the four regions of north-western Spain still inhabited by brown bears. The region is characterised by an oceanic climate with mild temperatures and high humidity, with annual mean temperatures ranging from 14°C on the coast, to 2–3°C on the highest points of the mountains (http://www.worldclim.org/). Asturias comprises 10,602 km^2^ with more than a million inhabitants distributed both in big cities and small towns from the coast to the Cantabrian Mountains. Population density is ca. 100 inhabitants/km^2^ (Instituto Nacional de Estadística http://www.ine.es/jaxiT3/Datos.htm?t=2886) and road density is 47.4 km/100 km^2^ (http://www.seap.minhap.gob.es/index.html). Elevation ranges from 0 to 2648 m a.s.l. (http://www.sadei.es/). The Cantabrian Mountains are principally covered by forests of chestnut (*Castanea sativa*), oak (*Quercus petraea*, *Q*. *robur*, *Q*. *pyrenaica* and *Q*. *ilex*) and beech (*Fagus sylvatica*), alternating with pastures and brushwood, and subalpine matorral above 1700 m [35,38].

### Bear occurrence data

We used a database of brown bear occurrences within Asturias that consists of geolocalized direct observations; indirect signs of the presence of the species (i.e., footprints, hair and scats); and damage records caused by bears to livestock, beehives, crops, and human activities and infrastructures. The database covers observations from 1995 to 2016 and has been compiled by the regional government of Asturias (S1 Fig). Observation data came from several sources, primarily: (a) systematic direct bear observations by regional government field staff (the Patrulla Oso, i.e., the Bear Patrol, and all the guards of the Principado), as well as by the Brown Bear Foundation (FOP, Fundación Oso Pardo), the Asturian Foundation for the Conservation of Wildlife (FAPAS, Fondo para la Protección de los Animales Salvajes) and personal observations of the authors; and (b) camera traps that were randomly located by the FAPAS and Bear Team during the last twenty years, mainly in forested areas where bears are less visible. Any indirect observations, i.e. tracks, signs and damages, were done by trained personnel. We removed observations with obviously erroneous or doubtful spatial locations (e.g., incomplete coordinates or poorly georeferenced observations).

### Definition of utilized brown bear range and identification of potential expansion areas

We evaluated the potential areas of bear expansion by using two different spatial scales [31]. We first defined a coarse scale of 5 x 5 km (25 km^2^), which is approximately the average size of Spanish brown bear home ranges [29,38,39]. For this coarse scale, we opted to model the suitability for distribution range rather than for occurrence [13], to obtain a primary and more general distribution of the favourable habitat for the species. Therefore, we first defined the distribution range of Asturian brown bears as pixels with 3 or more years of presence. We binarily classified the model into suitable and unsuitable areas. Within areas identified as suitable range from the coarse scale model, we then considered a model with a finer scale of 1 x 1 km to enable identification of suitable areas for brown bears within their area. For this scale, we therefore used raw observations as model input so that the model describes suitability for bear presence within areas that are regarded as within their suitable range.

### Environmental variables

We used 25 layers related to human infrastructures, vegetation and geomorphology as predictors in our habitat suitability models (S1 Table). Climatic variables came from the WorldClim 2.0 database (http://www.worldclim.org/) and were described by [40]. We used a discrete land cover layer available from the Cartografía Temática Ambiental del Principado de Asturias 1989–1998 (1:50,000), which we converted into percentage area within each pixel. We discarded any land cover class that did not possess at least 1% mean occurrence. We used each of the remaining classes as separate layers for our models, where each layer describes the relative abundance of a particular class. We also utilized the total number of land cover classes per pixel as well as the Shannon index of relative occurrences. We employed a Normalized Difference Vegetation Index (NDVI) as an index of greenness, which was provided by the Instituto de Recursos Naturales y Ordenación del Territorio (INDUROT). We used a digital elevation model from MDT200. From this model we derived elevation and mean aspect of the slopes. We obtained human population density from the Sociedad Asturiana de Estudios Económicos e Industriales (http://www.sadei.es/es/portal.do) and highway, road and footpath density as well as river density from geophysical layers maintained by the Principado of Asturias.

In case of a spatial correlation > 0.7 (Pearson coefficient) between two layers we only retained the layer that we regarded as most relevant for bear biology. For instance, we removed all climatic variables except seasonal variability in precipitation, since they were all correlated with elevation which is more ecologically relevant for bears [41,42].

We retained 19 uncorrelated variables: % shrublands, % gorse, % heath, % fern % forests, % pastures, % planted forest, % planted conifer forest % cliffs, mean value per pixel of the Shannon index of land cover heterogeneity and the normalized difference vegetation index, number of land cover classes, elevation, slope, precipitation seasonality, river, human population, highway, road and footpath density. All the variables were projected to the same reference system (ETRS89 / ETRS-UTM29) and scaled to a 5 x 5 and a 1 x 1 km resolution. For the final modelling, we removed any cells which had less than 50% of its area within the limits of the study region in each scale (most of these cells occurred along the coast) and we included all the variables mentioned above.

### Modelling with MaxEnt

**Fig 1:**
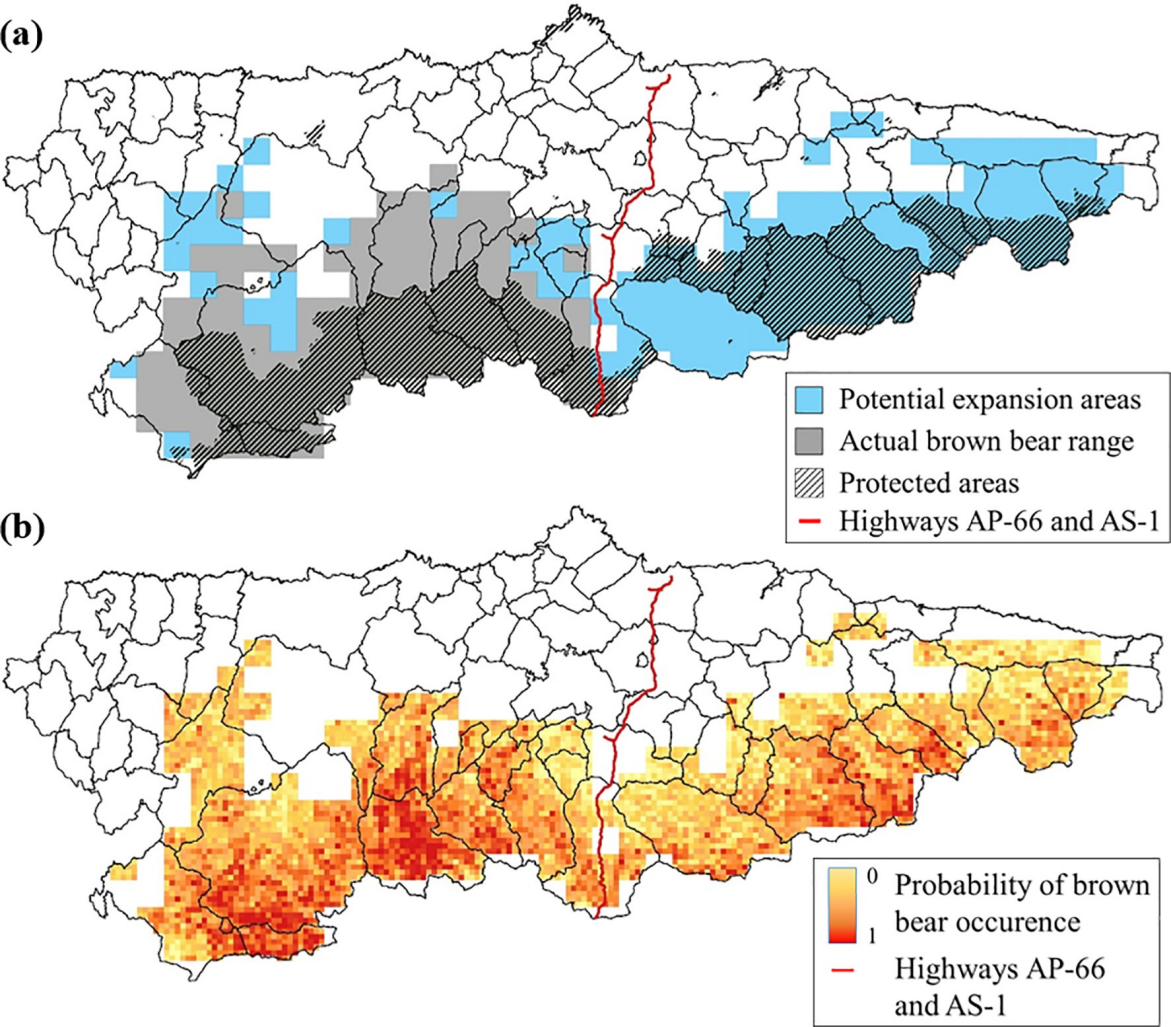
Coarse and fine scale models. Binary classification of a coarse (5 x 5 km) MaxEnt model broadly identifying suitable range areas, including favourable areas that are part of the current bear range, as well as protected areas (a); and model output from the fine scale (1 x 1 km) MaxEnt model identifying the probability of bear occurrence within the identified suitable bear range in the coarse model (b).

We used the software MaxEnt version 3.4.1 [15] called from the statistical environment R [43] version 3.3.3 using the packages dismo version 1.0–12 [44] and ENMeval version 0.2.0 [45]. Because of the limited number of observations in eastern Asturias, which could have been partly due to observer bias, we only trained models on data from east of the A-66 and AS-1 highways (Fig 1). These roads appear to function as partial physical barriers for the dispersal of brown bears between west and east subpopulations [31,34]. With this restriction, we retained 4878 bear locations which were clustered into 99 5 x 5 km pixels of bear range that were used as training locations for the coarse scale model, and 1091 1 x 1 km pixels within the suitable bear range which contained observations that were used for the finer scale model. For each scale, we utilized the centre coordinate of each cell, which was either classed as bear home range (coarse scale) or that contained a bear observation (finer scale), as occurrence data. We used 500 iterations, a convergence threshold of 10^−5^ and values from all cells in the west section of Asturias to build our first two models. Then, for both scales, we projected the model output onto the whole of Asturias once an optimal model structure had been selected (see below) using values from the eastern section of Asturias for the coarse scale and identified suitable range in the eastern part for the finer scale as background points.

### Selecting and evaluating the models

MaxEnt associates the presence data to environmental values using 5 different feature types (linear, quadratic, product, threshold and hinge), which represent different types of parameterizations. These feature types represent different transformations of the covariates, allowing the modelling of potential complex relationships between the variables and preventing overfitting [46]. The mean of each feature has to be close (within some error limits) to the empirical average over the presence locations [47]. The software also controls over-fitting using a regularization parameter which penalizes variables with low contribution to the model. Although machine learning algorithms such as the one used in MaxEnt generally favour more complex model solutions than likelihood based algorithms, overfitting can still be problematic since models essentially parameterize random spatial noise [48]. Hence, to identify an optimal model structure we evaluated candidate models with all types of feature combinations, each run over a set of regularization multipliers ranging from 0 to 10 (S2 Fig (13)). Each model included the same set of 19 uncorrelated environmental variables. We identified the best combination of feature types and regularization multiplier using Akaike’s Information Criterion corrected for small sample sizes (AIC_c_) [49]. We calculated the AIC_c_ values from the raw model output where the sum of the log transformed raw values were treated as equivalent to model likelihood [48]. Although potentially at odds with machine learning philosophy, AIC values have been shown to be an efficient and reliable method for identifying optimal levels of model complexity in MaxEnt applications. Following [50], we regarded models within 2 AIC_c_ units of each other as having equivalent empirical support. Of these models, we selected the model containing the least number of parameters, and if two models had the same number of parameters we chose the model with the lowest number of feature types. We regard this quantitative method of model selection beneficial, since it provides objective and rigidly defined criteria for evaluating the numerous alternative models associated with any given MaxEnt exercise.

To evaluate model performance we first used the AUC (Area Under a Receiver Operating Characteristic–ROC–Curve) value [51], which indicates how efficiently a model differentiates between occurrence and background locations. AUC values from 0.7 to 1 generally suggest that the model has adequate predictive ability [52]. Second, we calculated three model performance metrics based on cross validation using a checkerboard method to separate our occurrence data into training and testing [45]: AUC_test_ (ability of testing locations to distinguish between background and presence locations), AUC_diff_ (difference in the ability to distinguish between presence and background locations between training and test data [48] and OR_min_ (proportion of test locations with a value below the lowest value of training locations [45].

### Evaluating variable contributions

To evaluate the relative contribution of each environmental variable to the models we used a jacknife procedure and a heuristic method provided by MaxEnt. The jacknife test shows the gain in AUC value of each variable when used in isolation and the lack of gain when removed from the full set of variables (S3 Fig). The heuristic method calculates the percent contribution of each variable as the proportional contribution to the model training gain for every iteration of the model fitting process [14].

### Presentation of model output

For both models we used the complementary log-log (cloglog) format as model output, as it has an intuitive interpretation and is monotonically related to other potential output formats [15]. This format allows interpreting model output as a probability of occurrence. However, as we were interested in suitable areas for bear range in our coarse scale model, and not necessarily the relative suitability within identified range, we have presented the coarse scale model as a binary classification, which broadly identifies favourable bear range. For this purpose we used the 10 percentile training presence in the cloglog values as a threshold for suitable bear range. This threshold selects the value above which 90% of the training locations are correctly classified, and is one of the most common thresholds used in MaxEnt habitat suitability models [53]. However, we have presented the unclassed cloglog output of our coarse scale model in S4 Fig. For the fine scale we maintained the cloglog values in order to represent habitat suitability for bear occurrence.

### Identification of potential bear expansion areas and evaluation of the suitability of used range and expansion areas

For the coarse scale, cells classed as suitable but which were not part of the identified distribution range were regarded as potential expansion areas. To evaluate whether Asturian brown bears have expanded their range incrementally out of an initial core area (S5 Fig), we calculated the NODF nestedess index as an index of spatial nestedness over time [54]. This value describes the extent to which cells included in the range of a given year also form part of the range in subsequent years [14], which can be regarded as a temporal analogue to spatial nestedness [55]. Following Eriksson and Dalerum [14], we calculated the nestedness on annually identified pixels recognized as suitable range. The index value ranges from 100, indicating complete nestedness, to 0, indicating an anti-nested pattern. To evaluate if our observed values differed from random expectations we compared our nestedness index to values derived from 999 null models constrained to retain the original marginal sums. We only conducted this analysis on the coarse scale, as we do not regard absences of observations within pixels in the finer scale to be especially informative.

We evaluated whether suitability at the fine scale differed between the used range and the potential range expansion areas using a linear model. We used the log transformed raw model output as a response variable, and a 3 level factor as a predictor. This factor consisted of the classes obtained from the coarse model “used range” (suitable areas already occupied by bears), “expansion areas west” (unoccupied areas west of highways A-66 and A-S1) and “expansion areas east” (unoccupied areas east of highways A-66 and AS-1). We opted to separate the expansion areas in eastern and western Asturias because we suspect there may be observation bias in the eastern part, which may have underestimated the used range. We also added up to 7th order polynomials of the spatial coordinates to the model predictors to account for spatial autocorrelation. We selected this level of complexity for eliminating spatial autocorrelation by selectively adding polynomial complexity until we could not detect further autocorrelation using Moran’s I values calculated on the residuals [56]. For our data set, a polynomial approach was more efficient in removing spatial autocorrelation than approaches directly defining spatial autocorrelation in the model correlation matrix [57] or approaches using spatial eigenvectors as predictors [58]. To explore pairwise differences between the classes we used least square means with a Tukey correction for multiple comparisons [59,60].

## Results

### MaxEnt model selection and evaluation

The optimal coarse scale model identified by the AICc values included linear features, a regularization multiplier of 10, and discarded 12 variables of the whole set because of their limited contribution (S2 Table). The fine scale model included linear, quadratic, product and threshold features with a regularization multiplier of 0, and included all the selected variables with the minimum contribution being 0.6. Table 1 shows the different values of the evaluation metrics (see Methods) for the five candidate models for each of the coarse and fine scales with the highest empirical support. The best models at each scale had mean AUC values of 0.782 and 0.7368 respectively, showing adequate predictive ability. However, several models of both coarse and fine scale were regarded as having equal empirical support (Table 1). More complex models generally showed clear signs of over fitting, whereas less complex models lost predictive abilities (S2 Fig). The 10 percentile method yielded a threshold of 0.359 in cloglog values, which we used to defined suitable areas for bear range in the coarse scale.

**Table 1:** Evaluation metrics of the 5 candidate models with the highest empirical support at a coarse scale (5 x 5 km, a) and a fine scale (1 x 1 km, b), built to evaluate the suitability for brown bear range (coarse scale) and brown bear occurrence within suitable range (fine scale) within Asturias.

Model	Feature types^a^	Regularization multiplier	Full AUC	Mean AUC	AUC diff	OR min	AICc	Δ AICc	nparam
**Coarse scale**	L	10	0.795	0.782	0.010	0.031	1004	0	8
	L,Q,T	10	0.796	0.782	0.012	0.020	1005	0.20	9
	L,Q	10	0.796	0.782	0.012	0.020	1005	0.21	9
	L	7	0.796	0.786	0.011	0.031	1006	1.29	9
	L,Q,H	7	0.800	0.787	0.014	0.010	1006	2.14	12
**Fine scale**	L,Q,P,T	0	0.844	0.768	0.098	0.198	62041	0	299
	L,Q,H,P,T	0	0.844	0.769	0.085	0.177	62041	0	299
	L,Q,T	0	0.842	0.766	0.099	0.191	62119	78.41	292
	L,Q,H,T	0	0.842	0.768	0.085	0.182	62119	78.41	292
	L,Q,P,T	0.5	0.842	0.772	0.089	0.177	62161	119.88	272

^a^Feature types: L–linear, Q–quadratic, H–hinge, P–product, T–threshold

### MaxEnt variable contribution

The two variables that contributed the most to the coarse scale model (5 x 5 km) were altitude and slope (Table 2), which were confirmed both using the jacknife tests and the heuristic evaluations (S3 Fig and S2 Table). Both had a positive effect on the suitability for bear distribution (S6 Fig). Some variables of human impact (i.e., highways and footpaths) contributed modestly to this model with a negative influence on habitat suitability for bears (S5 Fig). Noteworthy is that several variables had no influence on the coarse scale model. These included precipitation seasonality, human population density, NDVI, pastures, cliffs, conifer plantations, rivers, shrubland, land cover classes and roads. The finer scale model (1 x 1 km) was most influenced by percentage of forest cover, precipitation seasonality and human population density (Table 2), with forest cover and precipitation seasonality being positively associated and human population density negatively associated with probability of bear presence (S7 Fig). As with the coarse scale model, the variable contributions were confirmed by both the jacknife tests and the heuristic evaluations (S3 Fig and S2 Table).

**Table 2:** The five most influential variables for coarse (5 x 5 km resolution) and fine scale (1 x 1 km resolution) MaxEnt models describing the probability of bear range and bear occurrence in Asturias, respectively. The percentage values are based on a heuristic method that estimates the proportional contribution of each variable to the model training gain for every iteration during model fitting.

Model	Variable	Percentage contribution
**Coarse scale**	Elevation	37.3
	Slope	34.4
	Fern	14.4
	Gorse	4.2
	Highways	3.1
**Fine scale**	Forest	24.3
	Precipitation seasonality	11.5
	Human density	10
	Slope	9.7
	Gorse	8.1

### Predicted range distribution and potential expansion areas

Brown bears occupy a total of 2430 km^2^ in Asturias. Most bear territory was identified in the south western part of the province, and our coarse scale model confirmed that most suitable areas are in the southern parts of the province (Fig 1A). The NODF nestdeness value characterizing the type of spatial expansion of brown bears in Asturias was marginally less nested than random expectations (NODF_obs_ = 18.42, NODF_exp_ = 18.70 ± 0.136, z = -1.97, p = 0.05). This suggests that the expansion has been not been caused by annual range expansions in which individuals have settled in neighbouring areas, but rather that they have dispersed in different directions and hence moving the utilized range over time.

Our coarse scale model identified 4977 km^2^ as suitable bear range, which represents close to half of the territory of Asturias (Fig 1A). However, most of the suitable areas in the western part of the province were already occupied (77% of identified areas, 2820 km^2^), 41.4% of them occurring inside protected areas (Fig 1A). This leaves relatively limited good areas for further expansion in this part of the province, although there might be more suitable areas in surrounding provinces. In the eastern part of the Asturias, however, 62% (2155 km^2^) of the land was classified as suitable, and this part of the province boasted 44.3% of the total areas identified as suitable bear range. Forty-one percent of all expansion areas (suitable areas outside the current bear range) were identified in protected areas (Fig 1A).

**Fig 2:**
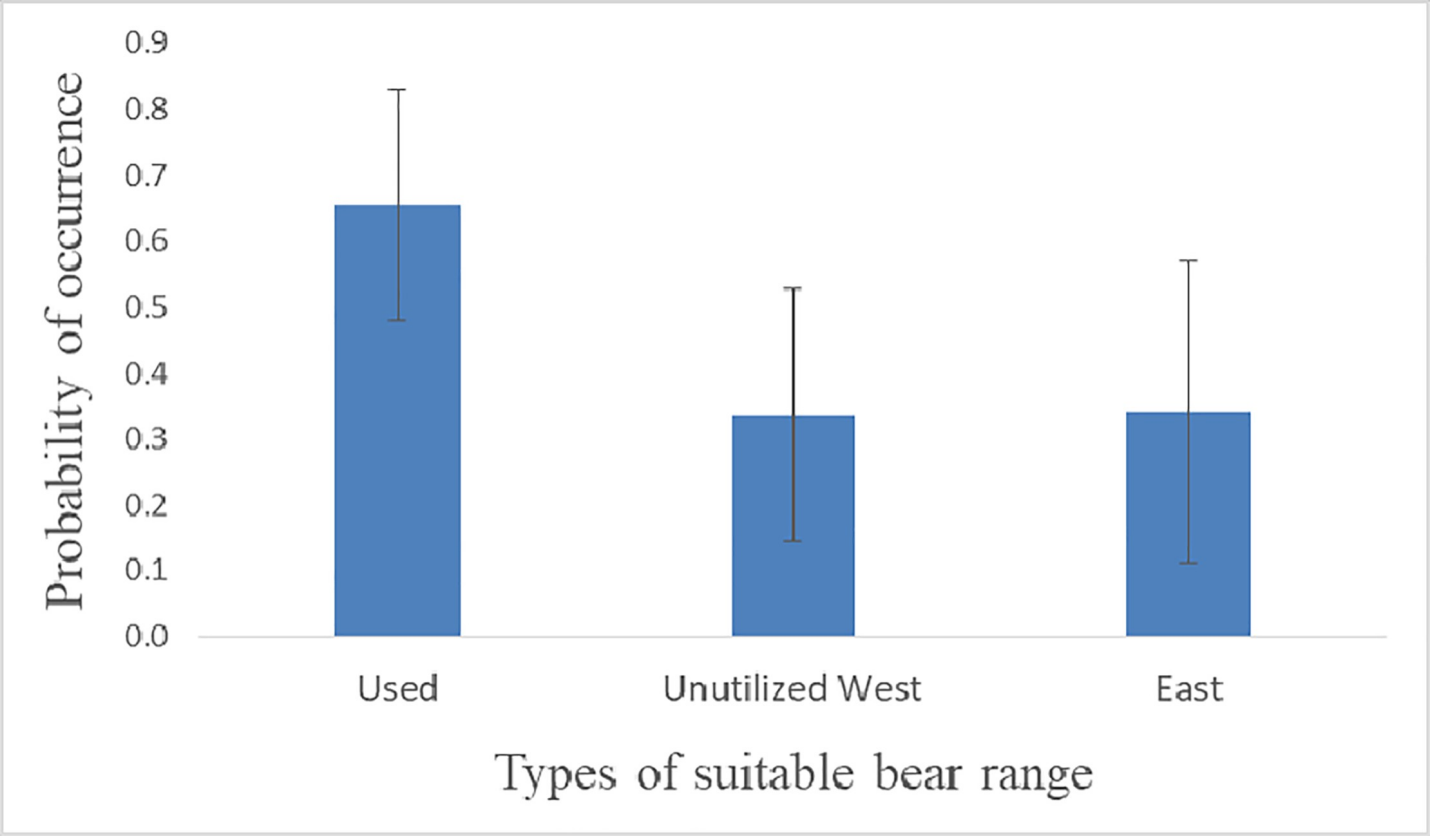
Probability of bear occurrence in 1 x 1 km cells inside the area identified as suitable bear range by a coarser (5 x 5 km) model, in utilised areas in western Asturias, unutilised areas in western Asturias, and eastern Asturias.

Our finer scale model identified the most favourable areas in the southwestern part of the range (Fig 1B). In line with this observation, there were significant differences in suitability between the used areas and the unused areas in the western and eastern parts of the province (F_2,2_ = 821.8, p < 0.001), with the utilised areas having significantly higher suitability than the unused areas both in the west (t = 26.7, df = 1, p < 0.001) and in the east of Asturias (t = 26.8, df = 1, p < 0.001). The areas in eastern Asturias had, however, higher suitability than the unused areas in the western part of the province (t = 26.8, df = 1, p < 0.001) (Fig 2).

## Discussion

Our study highlights that (a) a large portion of the Cantabrian Mountains belonging to Asturias is potentially suitable as bear range and (b) only most of the suitable western areas of the province have already been occupied. In fact, according to our coarse scale model, more than 75% of the favourable areas of western Asturias have records of brown bear presence. Because dispersal out of this area seems to be limited [34–36], if the population continues to expand as previously projected [33] it may either experience a local density increase or bears will expand into areas relatively far from the core of the population. Anecdotal information suggests that bears have already moved out of the most favourable areas and most of the observations made in cells outside of our classed bear range have been recorded in recent years.

Despite the consistent positive trend in the population size of the western subpopulation of the Cantabrian brown bear, the eastern subpopulation has had a substantially smaller population increase [35]. Although we cannot rule out that at least part of this lesser increase may be caused by observer bias, the stark contrast in population growth between the western and eastern subpopulations suggests high mortality rates in the eastern subpopulation and/or a relatively limited dispersal of bears out of western Asturias. Indeed, some previous studies have suggested a narrow connection between the two subpopulations [36,61]. Additionally, prior studies hypothesised a prevalence of low quality food-items in the eastern sector of the Cantabrian Mountains [39,62].

Our results also highlight the importance of using different scales to model habitat suitability. Indeed, the most important environmental variables differed between the coarser- and finer-scale models. This difference may be related to the relative influence of landscape features depending on the scale. While a broader spatial scale yields a general environmental description for the entire species distribution (even among different populations), a finer spatial scale is more related to local requirements [29]. On the one hand, altitude and slope were the most influential environmental variables in the coarse spatial scale model. This agrees with previous studies on brown bear distribution patterns [22,29,31,38,41,63,64]. Brown bears tend to appear in high and rugged areas, especially females with cubs trying to avoid infanticide. They also appear quite elusive nearby human settlements, in order to escape from disturbances produced by high human activity. Our results point to more inaccessible areas, where human density and activities are scarce, as suitable areas for the bears. Although with less influence, the coarse spatial scale model was also negatively affected by footpaths and highways. The latter has previously been negatively associated with habitat suitability for brown bears [22], suggesting that it may represent a limitation for brown bear dispersal.

On the other hand, forest cover (positive effect) and human population density (negative effect) were the most influential variables in the fine scale model. Indeed, forest cover represents crucial food and shelter for the Cantabrian brown bear, as one of its main food resources in the Cantabrian mountains are acorns [62,65,66]. On the other hand, human density may be related to bear avoidance of human disturbance, as proved in other studies showing that bears are more detectable further from human settlements, where human activity is greater [22,31,67,68].

We expect that the results of our models can also be used as a practical tool for brown bear damage prevention and conflict mitigation. Indeed, knowing the areas into which the population of the Cantabrian brown bear is likely expand in the near future would allow authorities and conservation organizations to focus information campaigns and pre-emptive damage control actions on these areas. Such proactive approaches are important for successful large carnivore conservation and management [69]. For Cantabrian brown bears, damages to apiaries are the main source of conflict, and damage prevention strategies have been shown to be effective to avoid them [70]. We suggest that our maps of potential brown bear range expansion areas should be overlaid with spatially explicit data on apiaries to allow for the identification of high risk areas where conflicts may occur. In addition, since brown bears have disappeared from certain areas some decades ago, local communities are no longer familiar with how to coexist with this large carnivore. Thus, local information campaigns directed at inhabitants of areas of potential bear expansion and based on studies like the present one may represent a crucial strategy to prevent human-wildlife conflicts.

## Supporting information

S1 Fig Brown bear occurrence data and location of the study area in Europe. (PDF)

S2 Fig **Evaluation metrics for 130 candidate models containing different levels of complexity defined by a range of five feature type combinations including linear (L), quadratic (Q), product (P), threshold (T) and hinge (H) features, each evaluated over a range of regularization multipliers ranging from 0 to 10, for (a) the coarse and (b) fine scales of the distribution of the Cantabrian brown bear in Asturias.** Evaluation metrics include delta AICc, which is the difference in AICc (Akaikes Information Criterion corrected for small sample sizes, calculated as the sum of the log transformed raw output penalized by the number of model parameters), AUC test, which is the AUC (area Under the receiving operator characteristics Curve) score for the testing data set, AUC diff, which is the difference in AUC scores between the training and testing data sets, and OR min, which is a threshold dependent statistic corresponds to the proportion of testing localities that have MaxEnt output values lower than the value associated with the training locality with the lowest value.
(PDF)

S3 Fig **Jacknife evaluations of variable contributions to the (a) coarse and (b) fine scale models.** The variables with the highest gain when used in isolation are slope for the coarse scale (a) and forest cover foir the fine scale model (b). These variables therefore seem to have provided the most useful information by themselves for each scale. The variables that decreased the gain most when omitted, and thus possessed the greatest amount of information not present in the other variables, were slope for the coarse scale (a) and population density for the fine scale model (b).
(PDF)

S4 Fig Output of the coarse scale model with a 5 x 5 km resolution. The map presents a clog-log transformation of the raw MaxEnt output, which can be interpreted as a probability of brown bear range occurrence.
(PDF)

S5 Fig **Schematic examples of incremental range expansion (a) out of an initial core area as well as (b) a patchy range expansion were no area is occupied two consecutive years,** their nestedness values as well as the association matrices used to calculate nestedness.
(BMP)

S6 Fig Associations between predicted suitability estimated from the coarse scale model each of the included environmental predictors. (PDF)

S7 Fig Associations between predicted suitability estimated from the fine scale model each of the included environmental predictors. (JPG)

S1 Table Description, source and original format of the 25 environmental variables initially developed for the construction of the models. Variables marked with * are the ones not correlated and ultimately used in the modelling.
(PDF)

S2 Table Variable contribution to the construction of the coarse and fine scale models. (PDF)

S3 Table Centre coordinates of the 5 x 5 km grids classed as bear home range used as bear occurrence data in the coarse scale model. (CSV)

S4 Table Centre coordinates of the 1 x 1 km grids that contained a bear observation used as bear occurrence data in the fine scale model. (CSV)
