# Supplementary figures and images for: Identifying potential areas of expansion for the endangered brown bear (Ursus arctos) population in the Cantabrian Mountains (NW Spain)
Source: PLoS One. 2019 Jan 4;14(1):e0209972. doi: 10.1371/journal.pone.0209972 (PMC6319805; doi:10.1371/journal.pone.0209972)

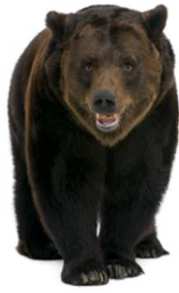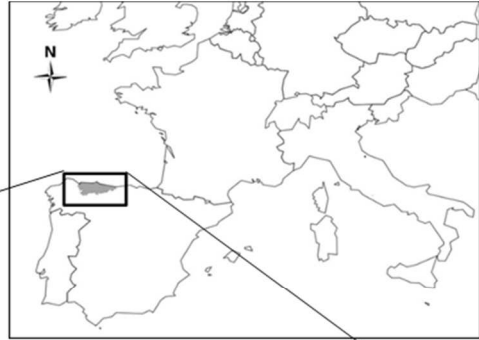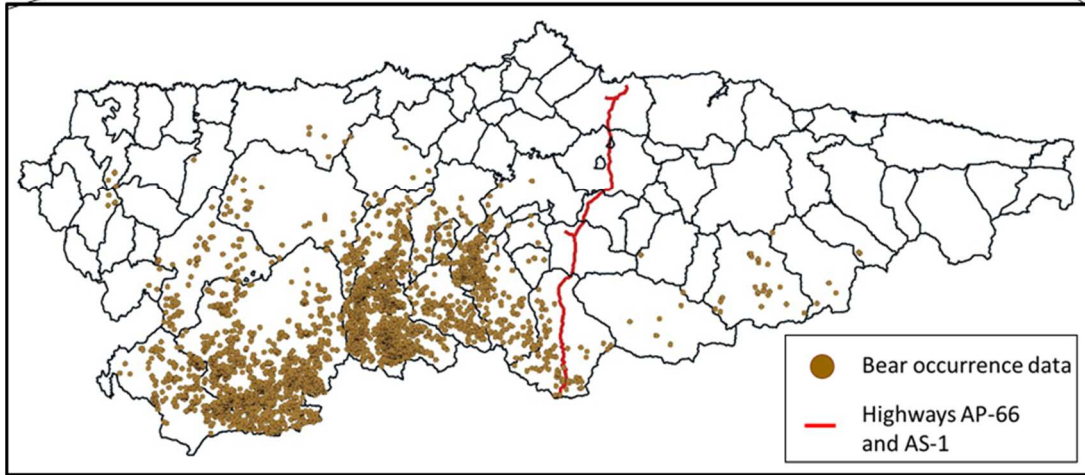

Supplement: S1 Fig — (PDF) [file pone.0209972.s001.pdf]

(a)

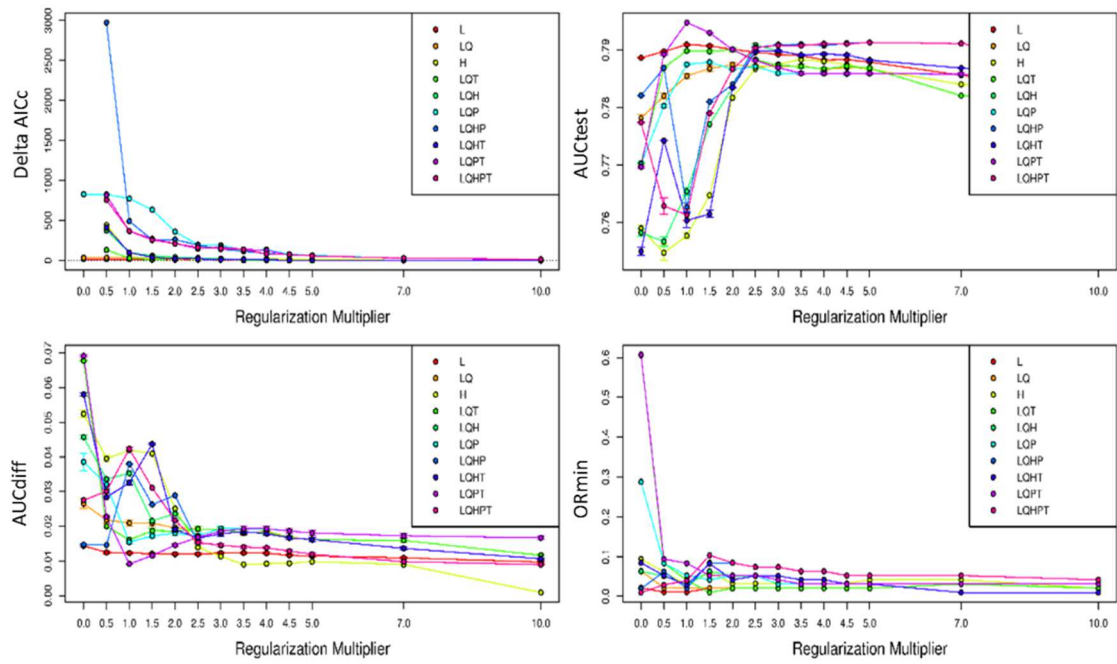

(b)

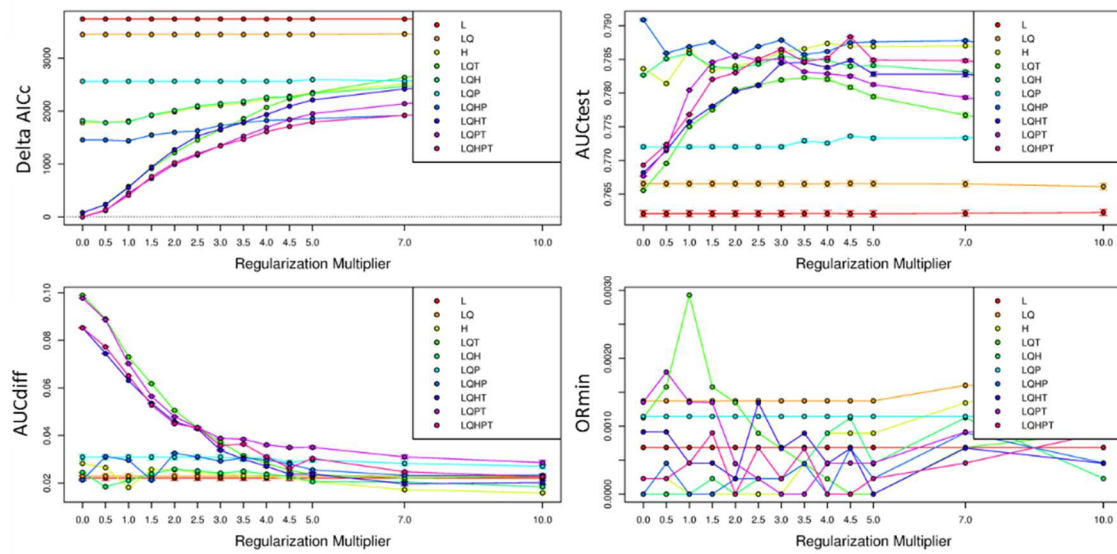

Supplement: S2 Fig — Evaluation metrics for 130 candidate models containing different levels of complexity defined by a range of five feature type combinations including linear (L), quadratic (Q), product (P), threshold (T) and hinge (H) features, each evaluated over a range of regularization multipliers ranging from 0 to 10, for (a) the coarse and (b) fine scales of the distribution of the Cantabrian brown bear in Asturias. Evaluation metrics include delta AICc, which is the difference in AICc (Akaikes Information Criterion corrected for small sample sizes, calculated as the sum of the log transformed raw output penalized by the number of model parameters), AUC test, which is the AUC (area Under the receiving operator characteristics Curve) score for the testing data set, AUC diff, which is the difference in AUC scores between the training and testing data sets, and OR min, which is a threshold dependent statistic corresponds to the proportion of testing localities that have MaxEnt output values lower than the value associated with the training locality with the lowest value. (PDF) [file pone.0209972.s002.pdf]

(a)

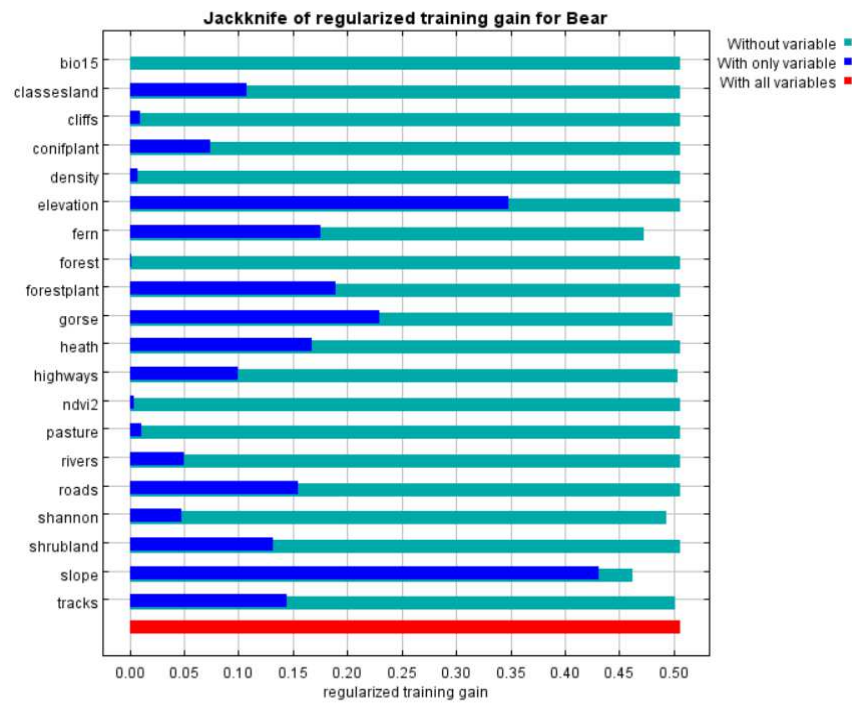

(b)

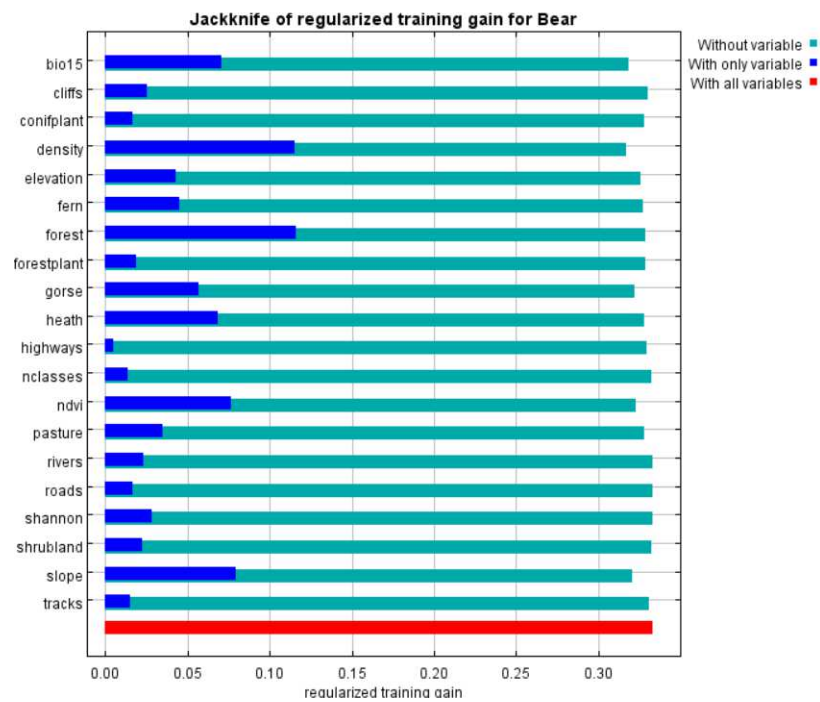

Supplement: S3 Fig — Jacknife evaluations of variable contributions to the (a) coarse and (b) fine scale models. The variables with the highest gain when used in isolation are slope for the coarse scale (a) and forest cover foir the fine scale model (b). These variables therefore seem to have provided the most useful information by themselves for each scale. The variables that decreased the gain most when omitted, and thus possessed the greatest amount of information not present in the other variables, were slope for the coarse scale (a) and population density for the fine scale model (b). (PDF) [file pone.0209972.s003.pdf]

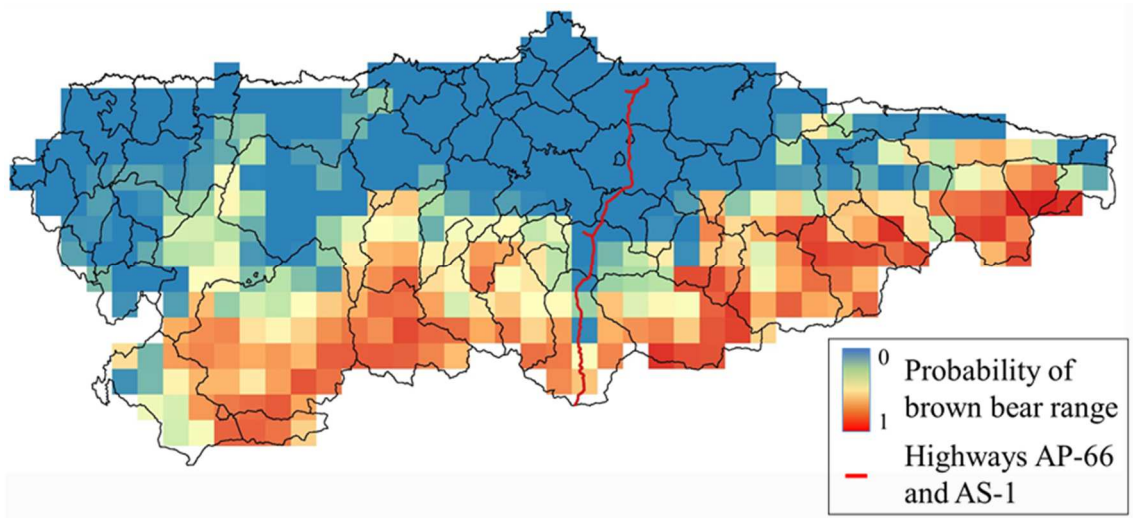

Supplement: S4 Fig — The map presents a clog-log transformation of the raw MaxEnt output, which can be interpreted as a probability of brown bear range occurrence. (PDF) [file pone.0209972.s004.pdf]

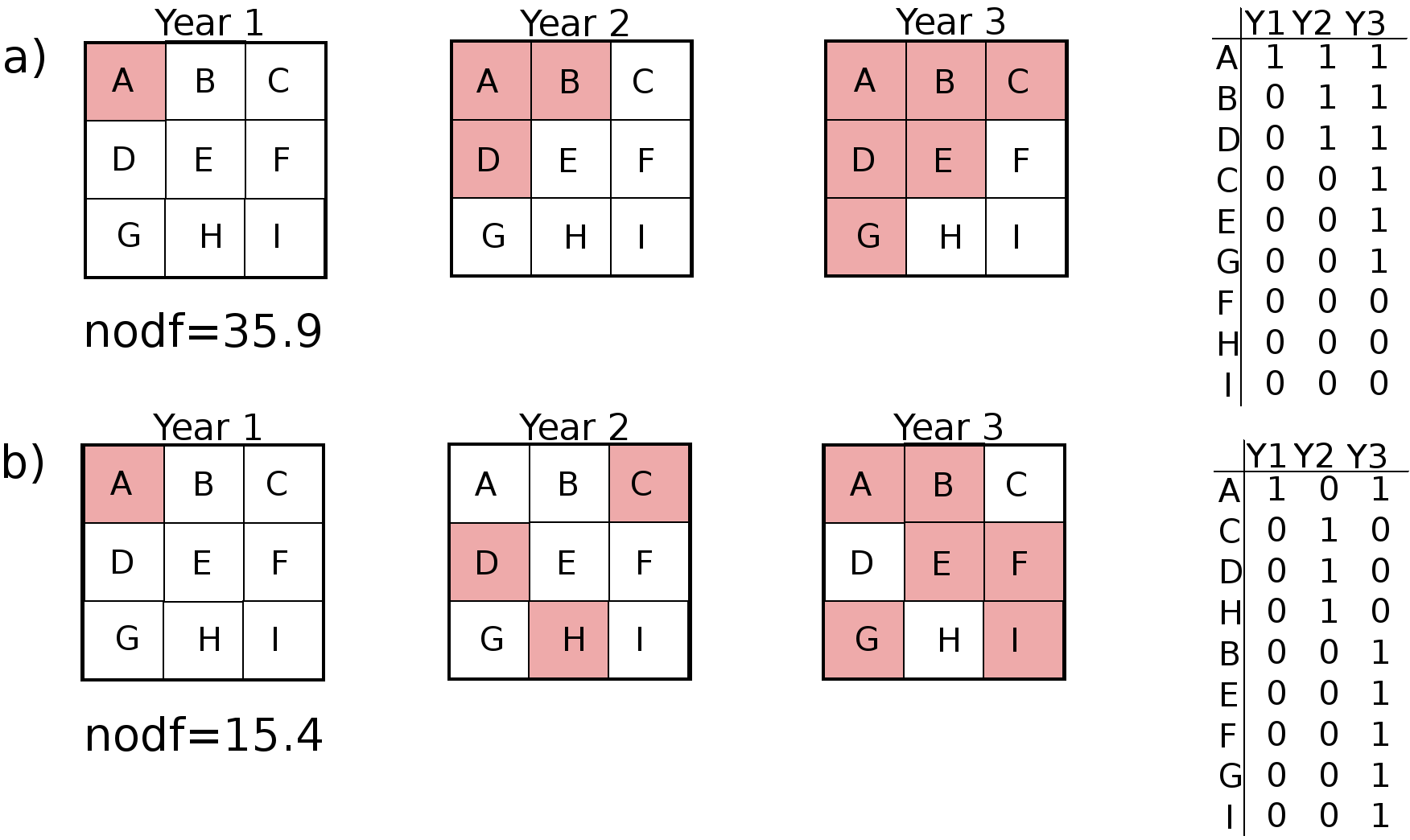

Supplement: S5 Fig — Schematic examples of incremental range expansion (a) out of an initial core area as well as (b) a patchy range expansion were no area is occupied two consecutive years, their nestedness values as well as the association matrices used to calculate nestedness. (BMP) [file pone.0209972.s005.bmp]

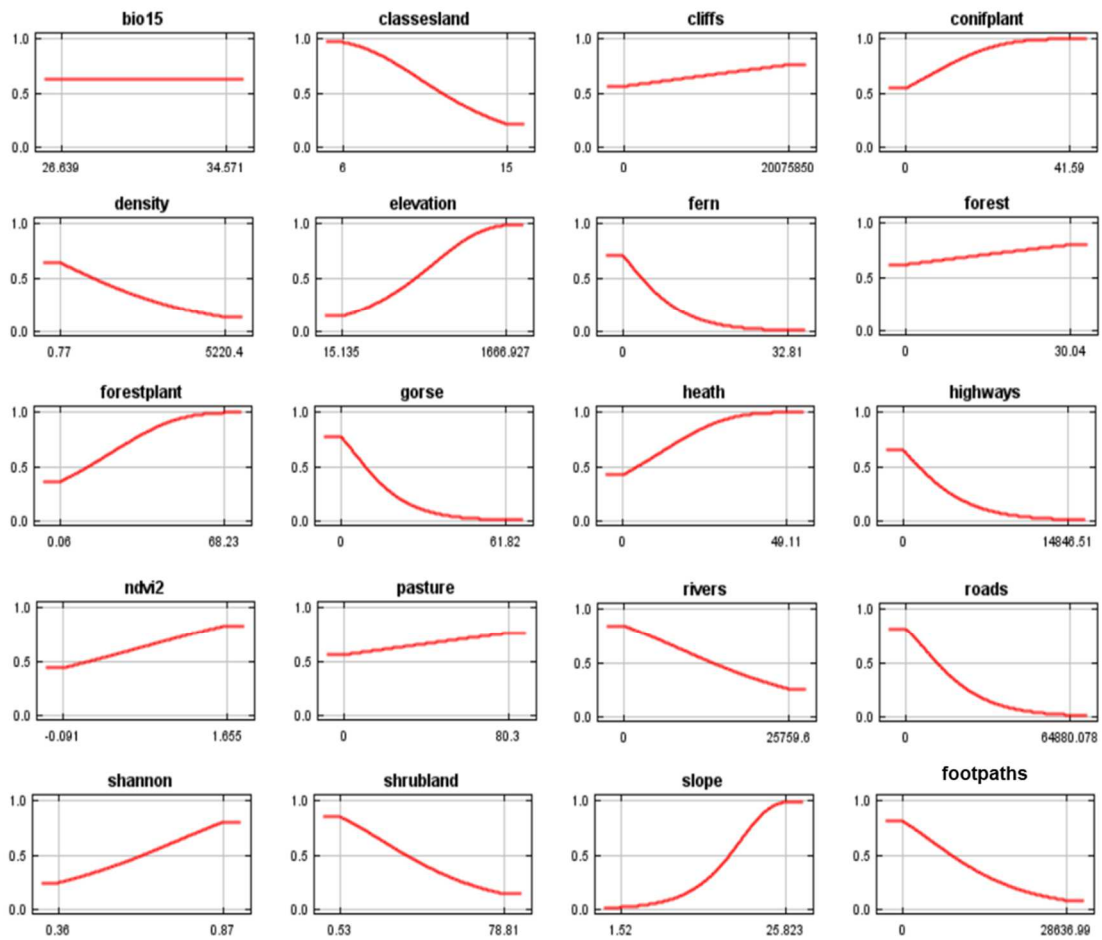

Supplement: S6 Fig — (PDF) [file pone.0209972.s006.pdf]

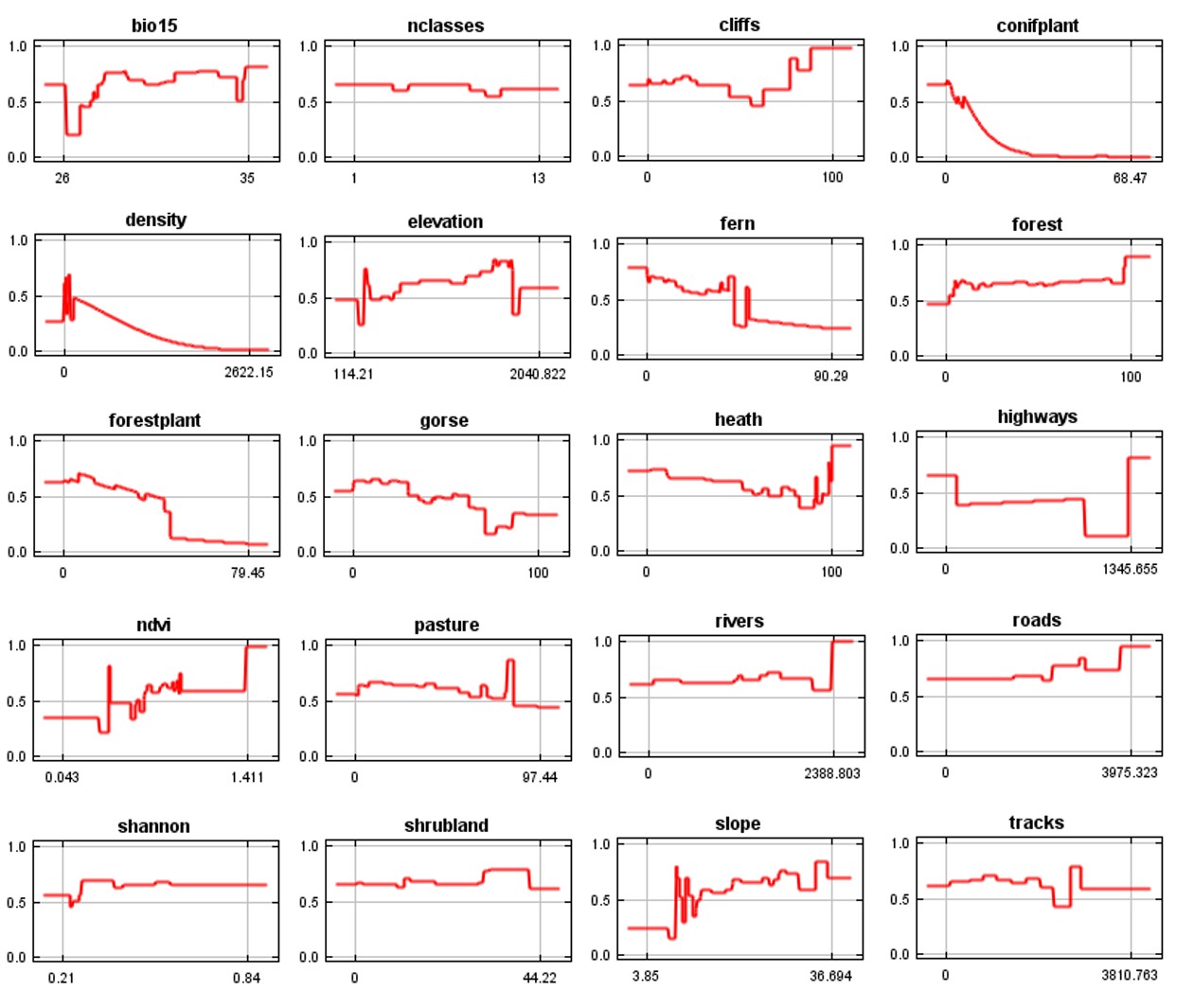

Supplement: S7 Fig — (JPG) [file pone.0209972.s007.jpg]
