# Supplementary material for: Identifying potential areas of expansion for the endangered brown bear (Ursus arctos) population in the Cantabrian Mountains (NW Spain)
Source: PLoS One. 2019 Jan 4;14(1):e0209972. doi: 10.1371/journal.pone.0209972 (PMC6319805; doi:10.1371/journal.pone.0209972)
Supplement: S1 Table — Variables marked with * are the ones not correlated and ultimately used in the modelling. (PDF) [file pone.0209972.s008.pdf]

Name	Description	Source	Format
Highways*	Length highways	BCN200	Vector
Roads*	Length autonomic and national roads	BCN200	Vector
Footpaths*	Length footpaths	BCN200	Vector
Rivers*	Length rivers	BCN200	Vector
Elevation and slope*		MDT200	Raster
NDVI*	Normalized difference vegetation index	Instituto de Recursos Naturales y Ordenación del Territorio (INDUROT)	Raster
Land cover*	% of each class per grid: cliffs, fern, forests, gorse, heath, pastures, conifer plantations, forest plantations, fruit plantations and shrublands, nº classes and Shannon index	Cartografía Temática Ambiental del Principado de Asturias 1989-1998 (1:50000)	Vector
Human density*		SADEI nomenclator	
Bio 1	Annual Mean Temperature	Worldclim	Raster
Bio 5	Max Temperature of Warmest Month	Worldclim	Raster
Bio 6	Min Temperature of Coldest Month	Worldclim	Raster
Bio 7	Temperature Annual Range (BIO5-BIO6)	Worldclim	Raster
Bio 12	Annual Precipitation	Worldclim	Raster
Bio 15*	Precipitation Seasonality (Coefficient of Variation)	Worldclim	Raster
