# Supplementary material for: Identifying potential areas of expansion for the endangered brown bear (Ursus arctos) population in the Cantabrian Mountains (NW Spain)
Source: PLoS One. 2019 Jan 4;14(1):e0209972. doi: 10.1371/journal.pone.0209972 (PMC6319805; doi:10.1371/journal.pone.0209972)
Supplement: S2 Table — (PDF) [file pone.0209972.s009.pdf]

Model	Variable	Percentage contribution
**Coarse scale**	Elevation	37.3
	Slope	34.4
	Fern	14.4
	Gorse	4.2
	Highways	3.1
	Shannon index	3
	Forest plantations	2.8
	Footpaths	0.8
**Fine scale**	Forest	24.3
	Precipitation seasonality	11.5
	Human density	10
	Slope	9.7
	Gorse	8.1
	Heath	7
	Fern	5.7
	NDVI	4.9
	Elevation	4
	Pasture	3.5
	Cliffs	1.8
	Forest plantations	1.6
	Conifer plantations	1.5
	Rivers	1.3
	Footpaths	1.1
	Highways	1
	Shrubland	0.9
	Number of landcover classes	0.8
	Roads	0.6
	Shannon index	0.6
